# Portrayal of electronic cigarettes in the news

**DOI:** 10.1002/puh2.84

**Published:** 2023-04-16

**Authors:** Chimwemwe Ngoma, Samar Mohamed Alhaj, Uchenna Frank Imo, Gabriel Ilerioluwa Oke, Yusuff Adebayo Adebisi

**Affiliations:** ^1^ Department of Journalism and Media Studies The Malawi University of Business and Applied Sciences Blantyre Malawi; ^2^ School of Medicine Ahfad University for Women, Omdurman Khartoum Sudan; ^3^ Department of Public Health University of Calabar Calabar Nigeria; ^4^ The Cognito Project Abuja Nigeria; ^5^ Global Health Focus Africa Abuja Nigeria

Dear Editor,

In recent years, e‐cigarette use has become a global phenomenon, with many countries witnessing a surge in the number of its users [1]. The estimated number of e‐cigarette users in 2018 was 58.1 million globally [1]. As the number of e‐cigarette users continues to rise, there is also a growing interest on the phenomenon by the media [2] with portrayals from different persuasions and perspectives. In general, they are either presented negatively or positively. Positive in a sense of being a potential less harmful alternative to smoking, or explaining its increasing popularity, and negative in terms of the regulatory challenges they present and the seemingly bad consequences on the part of the users. Given that approximately 80% of the public relying on general media for health information, more than the percentage that rely on health care professionals, the media plays a key role in informing the public about its various health dimensions [3]. In this article, we discuss the portrayal of e‐cigarettes in the news, its seeming implications and the need for balanced coverage.

The debate over e‐cigarette use continues among public health experts [4]. Some believe that e‐cigarettes offer significant benefits to public health, such as being a less harmful alternative to traditional smoking, whereas others hold mixed opinions and strongly oppose their use [4]. This is why portrayal is quite dichotomous; and mostly leaning toward the extreme ends of the continuum. These opposing views and division is probably one reason why many countries have implemented regulations regarding the sale and use of e‐cigarettes. For instance, the United States has introduced laws to restrict the marketing and sale of e‐cigarettes to minors, whereas some countries, like Brazil and Singapore, have banned their sale altogether [5]. In countries like the United Kingdom, electronic cigarettes are considered to be 95% less harmful than traditional cigarettes, making them a safer alternative for smokers [6] thus portrayed as such by the media. The United Kingdom is an example of a country which has taken a more welcoming stance toward e‐cigarette use as a tool for smoking cessation, with reputable public health agencies conducting independent research to scientifically validate their effectiveness. On the other hand, the World Health Organization (WHO) has a different stance by expressing its concern over the lack of regulation and potential health risks associated with e‐cigarettes [7] in many countries.

With the growing popularity of e‐cigarettes, it is crucial to continue exploring and understanding both its potential benefits and at the same time risks to contribute to a well‐informed public debate and discussion, at the same time addressing concerns related to their use. In countries where the media portrayal focuses on the negative aspects of e‐cigarettes, there is potential silencing of voices of those who have had positive experiences using them. It is important to strive for a balanced view and to inform policy that does not dismiss the potential benefits of e‐cigarettes as a smoking cessation aid. The media should allow for open discussion and research on e‐cigarettes, rather than pushing a one‐sided narrative. Different voices should be heard, without fear of being silenced, intimidated, or marginalized.

The media remains to have a significant impact on the public's perception of electronic cigarettes, particularly in the context of African countries where little research is being done. The portrayal of e‐cigarettes in the news can influence public opinion and shape government policies, as seen through the various policies countries have promulgated including banning or regulation. Moreover, the media remains a crucial source of information for individuals seeking guidance on health‐related issues. It is imperative to understand how and why the media portrays electronic cigarettes in certain ways in African countries, as this could help inform public health interventions and policies. By analyzing news portrayals of e‐cigarettes at an early stage of adoption and use, researchers can gain insight into the ways in which the media contribute and shape the ongoing debate over their regulation.

Various media portrayals of electronic cigarettes have varying consequences, including public panic and policy‐making that are not based on scientific evidence. The choice of news sources may influence how the issue is framed. A recent study comparing news coverage of e‐cigarettes in the USA, United Kingdom, and Korea found that the most prominent argument in the USA and United Kingdom was that e‐cigarettes are less harmful than combustible cigarettes, whereas Korean news focused on e‐cigarette ingredients [8]. These differences may be due to regional differences in priorities and research.

Research has shown that media coverage of electronic cigarettes tends to have emphasis on the opinions of government officials and lawmakers rather than those of health experts and electronic cigarette users [9]. As a result, news coverage may present a partial or skewed picture of the issue. This can lead to the sidelining of the views of vulnerable stakeholders, such as electronic cigarette users, who are not given an adequate platform to share their firsthand experiences with the benefits and negative consequences of using electronic cigarettes. News representation is often selective and influenced by political, economic, or ideological aims, according to some scholars. This suggests that people's perceptions of electronic cigarettes may be shaped more by the perspectives of social–political elites than the voices of consumers who have firsthand information about using electronic cigarettes. It is important for news coverage of electronic cigarettes to include a range of perspectives.

Health news is more likely to use alarm frames than coping frames [10]. Health issues like diseases are often depicted as killers, plagues, or hostile combatants in war, which can cause fear and vulnerability to the public. The use of such frames is sometimes attributed to the media's desire to gain public attention and maximize profit. This may conflict with the journalistic norm of objectivity. A mixed frame that features both high alarm information and high coping information is most likely to promote public health effects.

The portrayal of e‐cigarettes in the news requires a balanced view of its risk and benefits. Although some media coverage may overemphasize the potential risks and negative consequences, other news outlets may focus solely on the benefits and downplay the potential harm. It is important for journalists and media organizations to seek a diverse range of perspectives and sources, including those of health experts and electronic cigarette users, to provide a comprehensive and accurate picture of this issue. The media must continue to inform public debate and policymaking on electronic cigarettes in a more balanced and objective manner.

## AUTHOR CONTRIBUTIONS

This paper was conceptualized by Chimwemwe Ngoma and developed by Chimwemwe Ngoma, Samar Mohamed Alhaj, Uchenna Frank Imo, and Gabriel Ilerioluwa Oke. Chimwemwe Ngoma was involved in data acquisition and data interpretation. Chimwemwe Ngoma, Samar Mohamed Alhaj, Yusuff Adebayo Adebisi, Uchenna Frank Imo, and Gabriel Ilerioluwa Oke were involved in the drafting of the work and revising it critically for important intellectual content.

## CONFLICT OF INTEREST STATEMENT

The authors have either previously or currently been recipients of tobacco harm reduction scholarships awarded by Knowledge‐Action‐Change, a public health organization with a specific focus on tobacco harm reduction. The views and opinions expressed in the contents of this paper are the sole responsibility of the authors, and should not, under any circumstances, be regarded as reflecting the positions of Knowledge‐Action‐Change. Yusuff Adebayo Adebisi is an Editorial Board member of Public Health Challenges and a coauthor of this article. To minimize bias, he was excluded from all editorial decision‐making related to the acceptance of this article for publication.

## FUNDING INFORMATION

This paper did not receive any specific grant from funding agencies in the public, commercial or not‐for‐profit sectors.

## ETHICS STATEMENT

None.

## Data Availability

This is a Letter to the Editor and all sources used have been properly cited.
